# Survivin as a potential therapeutic target of acetylsalicylic acid in pituitary adenomas

**DOI:** 10.18632/oncotarget.25650

**Published:** 2018-06-26

**Authors:** Kinga Németh, Nikolette Szücs, Sándor Czirják, Lilla Reiniger, Borbála Szabó, Gábor Barna, Katalin Karászi, Péter Igaz, Vladimir Zivkovic, Márta Korbonits, Attila Patócs, Henriett Butz

**Affiliations:** ^1^ 2nd Department of Medicine, Faculty of Medicine, Semmelweis University, Budapest, Hungary; ^2^ National Institute of Clinical Neurosciences, Budapest, Hungary; ^3^ 1st Department of Pathology and Experimental Cancer Research, Semmelweis University, Budapest, Hungary; ^4^ Department of Laboratory Medicine, Semmelweis University, Budapest, Hungary; ^5^ MTA-SE Molecular Medicine Research Group, Hungarian Academy of Sciences and Semmelweis University, Budapest, Hungary; ^6^ University Clinical Centre, Belgrade, Serbia; ^7^ Department of Endocrinology, Barts and The London School of Medicine, Queen Mary University of London, London, United Kingdom; ^8^ MTA-SE “Lendulet” Hereditary Endocrine Tumors Research Group, Hungarian Academy of Sciences and Semmelweis University, Budapest, Hungary

**Keywords:** pituitary adenoma, nonfunctioning adenoma, cell cycle, survivin, acetylsalicylic acid

## Abstract

Acetylsalicylic acid (ASA) is known as a cancer preventing agent, but there is no data available regarding the effect of ASA on pituitary cells.

We investigated 66 nonfunctioning (NFPA) and growth hormone (GH)-producing adenomas and 15 normal pituitary samples. Functional assays (cell viability, proliferation, flow cytometry cell cycle analysis, caspase-3 activation and DNA degradation) were applied to explore the effect of ASA, YM155 (survivin inhibitor), survivin-targeting siRNA and TNF-related apoptosis-inducing ligand (TRAIL) in RC-4B/C and GH3 cells. Pituitary adenoma xenografts were generated in immunocompromised mice.

We found that survivin was overexpressed and TRAIL was downregulated in NFPAs compared to normal pituitary tissue. ASA decreased proliferation but did not induce apoptosis in pituitary cells. Additionally, ASA treatment decreased cells in S phase and increased cells in G2/M phase of the cell cycle. Inhibition of survivin using an inhibitor or siRNA-mediated silencing reversed the ASA-induced growth inhibition partially. In addition, we also found survivin-independent effects of ASA on the cell cycle that were mediated through inhibition of cyclin A, cyclin dependent kinase 2 (CDK2) and phospho-CDK2. We also aimed to test the effect of acetylsalicylic acid in an animal model using RC-4 B/C cells, but in contrast to GH3 cells, RC-4 B/C cells failed to adhere and grow a xenograft.

We concluded that ASA inhibited the growth of pituitary adenoma cells. Survivin inhibition is a key mechanism explaining its antineoplastic effects. Our results suggest that inhibition of survivin with small molecules or ASA could serve as potential therapeutic agents in NFPA.

## INTRODUCTION

Clinically nonfunctioning pituitary adenomas (NFPAs) constitute approximately 30-40% of pituitary adenomas [1, 2] with the majority being of gonadotrophic origin. While recurrent somatic mutations have not been identified in NFPAs [3], epigenetic alterations have been described both in oncogenes, such as *PTTG*, *POUF1*, *AKT2* and *HMGA1/2*. Further, tumor suppressor genes, such as *GADD45G*, *MEG3*, *PLAGL1* were found to be hypermethylated [4]. Alteration in cell cycle regulators, such as overexpression of cyclins (CCNA1, B1, B2) and decreased expression of cyclin dependent kinase inhibitors (CDKN1A, CDKN2A, CDKN1B) are found in most pituitary adenoma types [4]. We previously identified microRNA-induced altered G2/M transmission in NFPAs due to decreased expression of WEE1 kinase and increased level of CDC25A in pituitary adenomas [5, 6].

Beside cell cycle alterations, inhibition of apoptosis is also a potential mechanism leading to tumorigenesis in pituitary adenomas. This could occur due to overexpression of *GAL3* and *BAG1*, or decreased expression of *DAPK1*, genes known to regulate apoptosis. Proteomic studies also suggest deranged apoptosis in pituitary adenomas [7].

Survivin, a member of inhibitor of apoptosis protein (IAP) family, plays a role in both apoptosis inhibition and cell cycle regulation, although the mechanism of its action has not been clarified. Survivin, together with AURKB and INCENP, forms the chromosomal passenger complex, a key regulator of mitosis. Elevated survivin expression is present in a variety of cancers [8] and its expression correlates with aggressiveness and poor survival [9, 10]. Thus, survivin is considered as a potential target in cancer therapies [11]. In our previous study we performed gene expression profiling of G2/M transition regulators in NFPAs compared to normal pituitary (NP), and showed that survivin (*BIRC5*) and Aurora kinase B (*AURKB*), were the most upregulated genes [6]. However, little is known about the role of survivin in pituitary tumors. In a recent paper published while our work was in final preparations, the authors showed that survivin associated with invasiveness of pituitary adenomas [10, 12]. As survivin is suppressed by acetylsalicylic acid (ASA), an agent used as chemoprevention for colorectal cancer [13, 14], we studied the therapeutic potential of ASA in pituitary tumours, and assess its potential effects on survivin. We hypothesized that ASA might affect various cellular processes through survivin, that could lead to reduced growth and beneficial overall effects.

## RESULTS

### Survivin is overexpressed in nonfunctioning and GH-producing pituitary adenomas

In our previous study performed on 34 NFPA and 10 normal pituitary tissue specimens we found that survivin was the most upregulated gene at the mRNA level (fold change: 5.1; p=0.0004) in NFPA [6]. Now, we extended this study to GH-producing adenomas with 12 samples and we found that survivin mRNA was overexpressed compared to normal pituitary (fold change: 4.4; p=0.0060).

**Figure 1:**
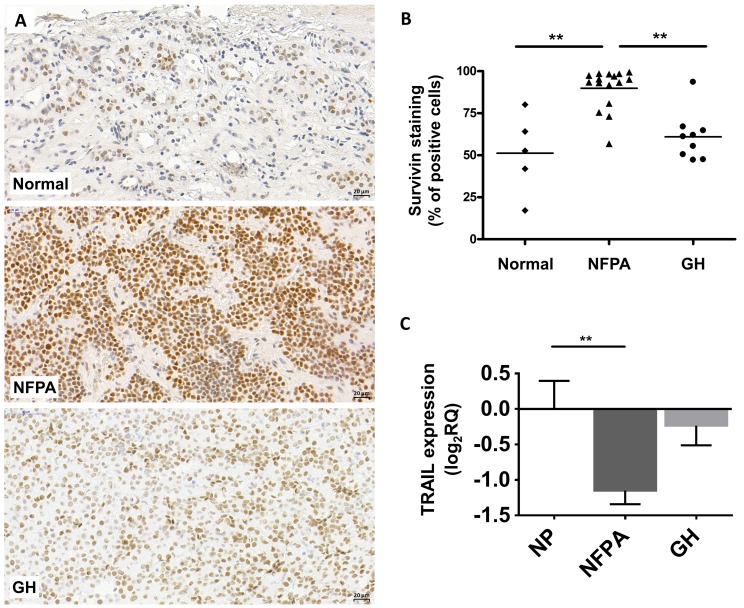
Representative images for survivin immunostaining on pituitary adenomas **(A)** Survivin staining localizes in cell nuclei. *Up:* normal pituitary, *middle:* NFPA, *bottom:* GH-secreting adenoma. **(B)** Survivin staining score showing increased survivin protein abundance in NFPA tissues compared to normal tissues. **(C)** TRAIL mRNA expression in pituitary adenomas (NP n=10; NFPA n=29; GH n=12) (^**^: p<0.01).

Next, we investigated survivin protein expression in NFPA and GH-secreting pituitary adenomas. Using immunohistochemistry staining, we detected only nuclear survivin staining in normal pituitary (n=5), NFPA (n=16) and GH-producing (n=9) pituitary adenoma tissues. No cytoplasmic staining was observed. Survivin protein was overall overexpressed in NFPAs (p=0.025) compared to normal pituitary (Figure 1A-1B). Analyzing moderate and strong positivity we found that 76.9% (13/16) of NFPA samples showed higher survivin protein expression compared to normal pituitary specimens (p=0.028).

Interestingly, despite of significantly elevated mRNA level in GH-secreting adenomas, survivin protein increase did not reach significance compared to normal pituitary (p>0.05) (Figure 1A-1B).

Survivin mRNA and protein expression did not show significant correlation with Ki67 index in NFPA and GH-producing pituitary adenoma tissues.

As survivin inhibits apoptosis induced by TNF-related apoptosis-inducing ligand (TRAIL) and ASA enhances and sensitizes cells to TRAIL-induced apoptosis in different cell lines and xenograft models [15, 16], we investigated TRAIL expression in pituitary adenoma. TRAIL was downregulated in nonfunctioning pituitary adenoma compared to normal pituitary (Figure 1C).

### ASA reduces cell growth mainly by inhibiting proliferation and cell cycle and not by apoptosis induction in pituitary adenoma cells

**Figure 2:**
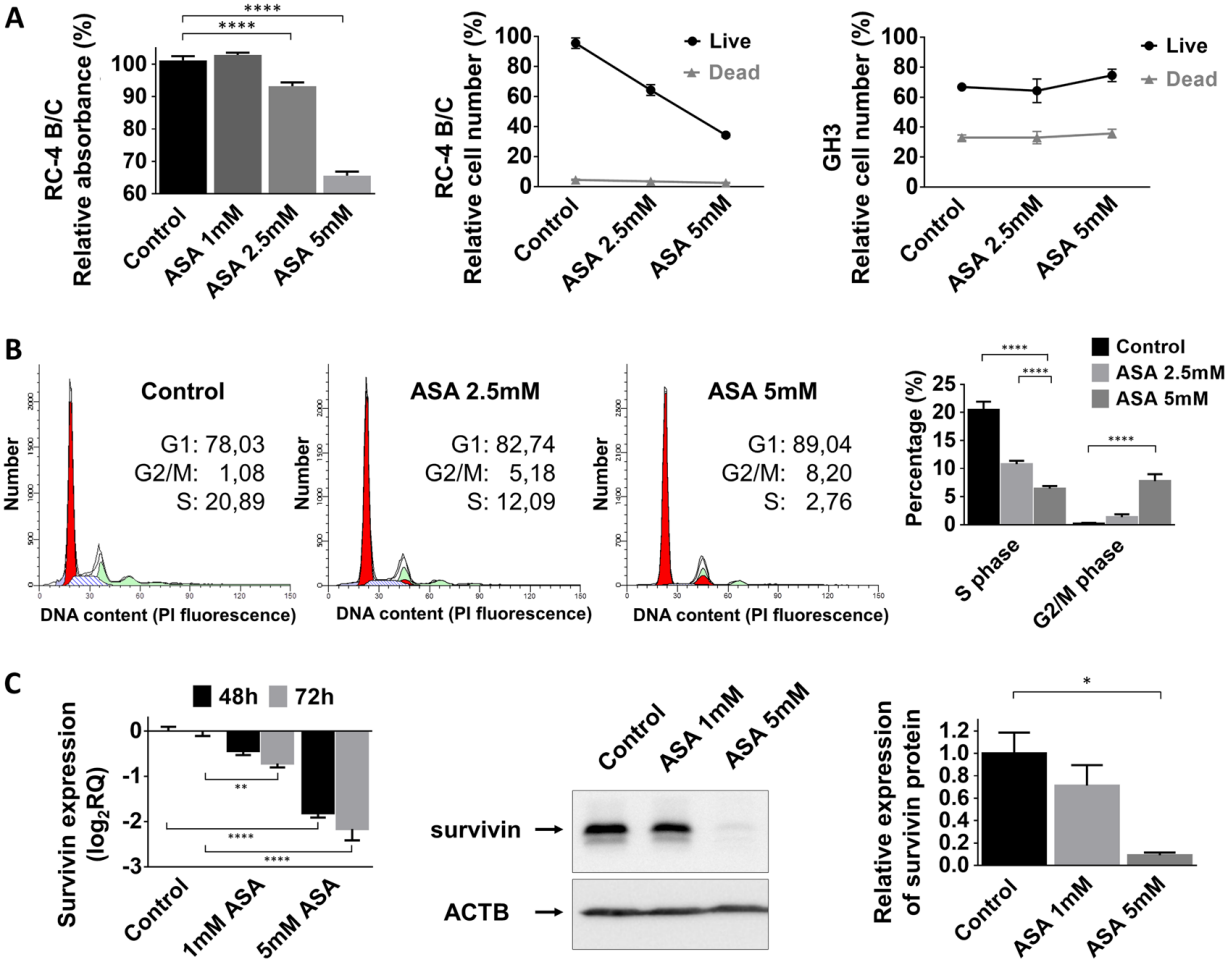
Effect of ASA treatment on pituitary adenoma cells **(A)** Cell proliferation in RC-4 B/C cells but not GH3 cells decreased after 2.5 and 5 mM ASA treatment **(B)** Cell cycle analysis using flow cytometry in RC-4 B/C cells showed decrease in S phase and increase in G2/M phase population upon ASA treatment. **(C)** Survivin mRNA and protein expression decreased after ASA treatment. ^*^: p=0.013; ^****^: p<0.0001.

Next, we investigated the influence of acetylsalicylic acid on pituitary adenoma cell viability, proliferation and cell cycle progression. ASA significantly decreased cell viability in a dose-dependent manner in the gonadotropin-secreting RC-4 B/C cell line but not in the growth hormone-secreting GH3 cells (Figure 2A). Additionally, cell proliferation was also decreased to 64±10.8% by 2.5 mM (p<0.0001) and to 44±3.9% by 5 mM (p<0.0001) ASA treatment in RC-4 B/C cells. In GH3 cells, we could not demonstrate a similar inhibitory effect (Figure 2A).

Next, we investigated whether this inhibitory effect could be linked to changes in cell cycle. Using flow cytometry cell cycle analysis, we observed a decreased number of cells residing in S phase (20.5% of untreated cells versus 10.8% for 2.5 mM (p=0.004) and 6.5% for 5 mM (p<0.0001) ASA treatment). The percentage of cells accumulating in G2/M phase was also increased upon ASA treatment (0.2% of untreated cell versus 1.4% for 2.5 mM (p=0.92) and 7.8% for 5 mM (p=0.0005) ASA) (Figure 2B).

Published data indicate that beside growth inhibition ASA can also induce apoptosis in several tumor cell types. However, using Trypan blue staining we could not detect an increase in the number of dead cells following ASA treatment (Figure 2A). In accordance with these data, we also could not demonstrate enhanced DNA degradation or increased Caspase-3 activation after 2.5 mM or 5 mM ASA treatment (Supplementary Figure 1A-1B). Because ASA can specifically sensitize tumor cells to apoptosis we evaluated if ASA might be able to augment TRAIL-mediated apoptosis. However, we found that in pituitary cell lines TRAIL was not able to induce apoptosis and ASA could not sensitize for TRAIL treatment compared to the positive control (Supplementary Figure 1C).

### Survivin downregulation is not the exclusive mechanism mediating the effect of ASA on pituitary adenoma cells

We hypothesized that the ASA-related effect on cell proliferation in pituitary adenoma cells might be linked to survivin. We found that 1 mM and 5 mM treatment with ASA gradually decreased survivin mRNA and protein expression in RC-4 B/C cells (Figure 2C).

**Figure 3:**
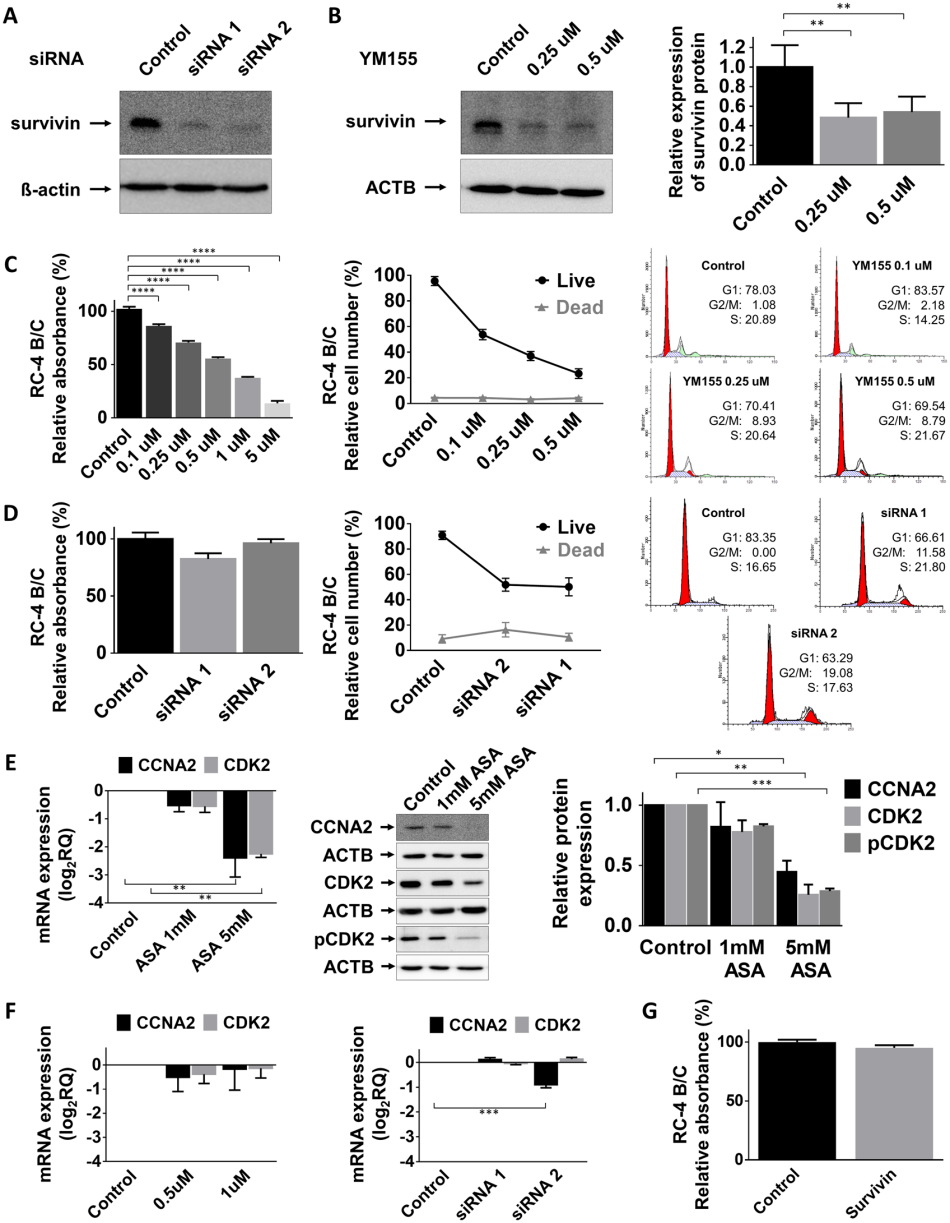
Effect of survivin inhibition **(A)** Survivin silencing by siRNA on RC-4 B/C cells. **(B)** Survivin inhibition by YM155, a small molecule inhibitor in RC-4 B/C cells. The effect of YM155 **(C)** and survivin siRNA **(D)** on pituitary adenoma cell viability, proliferation and cell cycle phases of RC-4 B/C cells (see details in the text). **(E)** Decrease of CCNA2, CDK2 mRNA and CCNA2, total CDK2 and p-CDK2 protein abundance following ASA treatment in RC-4 B/C cells. *Left:* mRNA, *middle* and *right*: western blot and densitometry. **(F)** YM155 and survivin siRNA transfection did not significantly alter CCNA2 and CDK2 expression in RC-4 B/C cells. **(G)** Survivin overexpression did not reduce the viability of RC-4 B/C cells. ^*^: p<0.05; ^**^: p<0.01; ^***^: p<0.001; ^****^: p<0.0001.

In order to demonstrate that ASA specifically acts through survivin we inhibited survivin using a specific small molecule inhibitor, YM155 as well as silencing with a specific siRNA. We found that both YM155 and survivin siRNA led to dramatic survivin protein decrease in RC-4 B/C cells (Figure 3A-3B). Using the pharmacologically relevant concentrations (0.1-5 μM) of YM155 we detected significant reduction of proliferation of RC-4 B/C cells (Figure 3C) but not in GH3 cells (Supplementary Figure 1D). It is noticeable that in higher concentrations (25, 50 and 100 μM) GH3 cell proliferation was also decreased (data not shown). Similarly, survivin-specific siRNA treatment did not significantly decrease cell viability (Figure 3D). Regarding the cell cycle, unlike ASA, treatment with YM155 survivin inhibitor resulted in increased accumulation of cells in G2/M phase but not a decrease in S phase (Figure 3C-3D). Together, siRNA knockdown of survivin caused decreased cell growth and increased number of cells in G2/M (Figure 3D).

As both ASA and survivin inhibition (siRNA and YM155) increased the number of cells accumulating in G2/M phase but only ASA decreased the percentage of cells in S phase, we investigated how the treatments affected G1/S checkpoint regulators (CCNA2, CDK2 and p-CDK2). We demonstrated that CCNA2, CDK2 and p-CDK2 were all decreased after ASA treatment (Figure 3E) while YM155 treatment or survivin siRNA transfection did not alter these (Figure 3F).

Next, to further investigate survivin’s function, we transfected RC-4 B/C cells with a survivin-expressing vector and assessed cell proliferation. However, survivin overexpression did not have any effect on cell proliferation measured by Alamar Blue proliferation assay (Figure 3G).

### Generation of a gonadotroph pituitary adenoma xenograft model in mice

Based on our *in vitro* results we decided to generate a pituitary adenoma animal model using RC-4 B/C cell line to test the effect of per os ASA on tumor formation and tumor growth. RC-4 B/C xenograft has been never generated before, therefore we tried several different conditions. We injected severe combined immunodeficient (SCID) mice with gradually increasing cell number (5-20x10^6^ cells/injection). In a preliminary experiment tumor cells were injected in 0.2 ml of growth medium, but tumor formation did not occur in three weeks. Next, we mixed cells with Matrigel before injection, and found that injected cells were palpable after 20x10^6^ cells injection only in the first two weeks. However, they were not able to adhere and grow and they absorbed progressively in the following weeks (Supplementary Figure 2). We used GH3 cells as a positive xenograft control as it has been described in literature. As *in vitro* ASA did not have an effect on GH3 cells we disregarded per os ASA treatment *in vivo*.

## DISCUSSION

Our data suggest that ASA decreases cell viability and proliferation of pituitary adenoma cells, as reduced number of cells are in S phase with cells stalling at the G2/M checkpoint. We investigated the mechanism behind this phenomenon hypothesizing that ASA inhibits survivin, a molecule overexpressed in NFPAs. ASA treatment resulted in proliferative and viability changes that resembled the effects of pharmacological (small molecule survivin inhibitor) or genetic (survivin-targeting siRNA) inhibition of survivin. However, analyzing cell cycle phases we observed slight differences. While both ASA and survivin inhibition led to an increase in cells in G2/M, only ASA caused a decrease in the number of cells in S phase. Cyclin A2-CDK2 complex has an important role during S phase progression [17]. ASA treatment resulted in significant cyclin A2 and total/phospho-CDK2 decrease, but these effects were not seen after YM155 treatment and survivin siRNA transfection. In line with this a previous publication claimed, that survivin inhibition leads to G2/M arrest in prostate cancer cells [18]. Based on these results we may conclude that ASA can act both through and independently from survivin on pituitary cell growth.

The effect of ASA on apoptosis is cell type specific [19, 20], as some cells (e.g. HT-29 colon adenocarcinoma cells) do not show this phenomenon [21]. There are several suggested mechanisms for the apoptotic effect of ASA [20], such as induction of caspase activation, inhibition of NF-Κβ and p38 MAP kinase activation [16], BCL2 downregulation, induction of BAX translocation into the mitochondria [20] and TRAIL downregulation [6]. We found that TRAIL was reduced in NFPA tissues. Taken the facts that survivin inhibits TRAIL-induced apoptosis [22] and ASA can sensitize cells to apoptosis induction [15] and can downregulate survivin, the expression pattern of these two genes in pituitary adenoma tissues raised the possibility that ASA may exert an apoptosis-inducing effect on pituitary adenoma cells. Interestingly, we could not confirm an apoptosis-inducing effect of ASA in pituitary cell lines using three different apoptosis detection methods. Other publications have also revealed that in contrast to other nonsteroidal anti-inflammatory drugs, the apoptosis-inducing effect of ASA is not present in every cell type [20].

Survivin was found to be overexpressed in most human cancers independently of mitotic index [23]. Interestingly, survivin expression was demonstrated not only in malignant tumors, but in low-grade, benign tumors of the central nervous system [24]. In pituitary adenoma, we could not show correlation between Ki67 index and survivin expression. Overexpression of survivin by vector construction in pituitary adenoma cells did not increase further proliferation indicating that survivin on-off expression state is probably more important than the extent of overexpression. This is supported by a recent publications where the authors suggested that survivin overexpression was potentially associated with pituitary adenoma invasiveness [10].

Regarding cellular localization, cytosolic survivin was suggested to function as apoptotic suppressor while nuclear survivin is related to cell division regulation [23]. In line with these data we detected nuclear survivin staining in NFPA and found survivin acting on cell proliferation but not on apoptosis in functional *in vitro* experiments. Our results are similar to those published by Wasko et al., 2009 and Jankowska et al., 2008 who also found nuclear staining in pituitary adenomas [24, 25]. To investigate the function of survivin in pituitary adenoma cells we chose to use the small molecular weight inhibitor, YM155, because similar to acetylsalicylic acid, it has an effect on survivin transcription. Survivin transactivation requires the binding of a transcription factor complex to the promoter. YM155 inhibits Sp-1 and ASA inhibits E2F-1 binding to the survivin promoter, hence both agents inhibit the formation of the transactivator complex. YM155 induces apoptosis in p53-deficient cancer cells, and it was shown to be effective in *in vivo* models of different cancers [11, 23]. Furthermore, this molecule is under clinical trials for the treatment of different cancers [26, 27]. Inhibiting survivin by either YM155 or siRNA-mediated silencing mimicked the effect of ASA, indicating that survivin is a promising target in the treatment of NFPAs.

We attempted to generate a gonadotroph pituitary adenoma xenograft using the rat RC-4 B/C cell line, which is the only commercially available gonadotropin-secreting cell line available, in order to test the effect of per os ASA on xenograft growth. Such a model has never been published before. Despite adjusting experimental conditions, the RC-4 B/C xenografts failed to adhere and grow, in contrast to GH3 xenografts, precluding us from performing *in vivo* studies to confirm the effects of ASA.

It is important to find novel therapeutic targets in NFPAs, where surgery and radiotherapy are the only therapeutic options. Due to the lack of specific hormone excess causing prominent signs and symptoms, these benign tumors are usually discovered when they have already caused optical nerve compression, other mass effect-associated symptoms, or invaded into the surrounding sinuses. At this point, the success of surgical removal is not always guaranteed. In the case of NFPAs, both ASA and survivin inhibitors could be promising options. ASA has the advantage of being widely used for thrombosis prevention, as analgesic and anti-inflammatory agent.

The only concern with ASA as a potential therapeutic agent in pituitary adenomas is the risk of pituitary apoplexy. There are reports suggesting anticoagulant therapy as relative contraindication in patients with known macroadenoma [28]. However, patients with macroadenoma who had pituitary apoplexy received aggressive anticoagulative and antiplatelet therapy (ASA combined with clopidogrel and full-dose enoxaparin), therefore, it is unclear if ASA administration as a single therapy would increase the risk of apoplexy [28, 29]. Semple et al. concluded that the most common triggers for pituitary apoplexy are pituitary stimulation, surgery, particularly coronary artery surgery and coagulopathy [30]. This observation was further confirmed by Vargas et al., who could not confirm the role of antiplatelet therapy in pituitary apoplexy by bivariate and multivariate analysis [31]. Taken together, it is possible that ASA will not elevate the risk of pituitary apoplexy, however this requires further investigation.

ASA is an effective, well-tolerated, cheap option especially compared to other chemotherapeutic drugs. Naturally, the antineoplastic and/or preventive effect of ASA on NFPAs requires further studies, yet it still seems a promising and relatively harmless option.

In summary, our results show that survivin is overexpressed in NFPAs but not in GH-secreting adenomas. ASA reduces cell viability and proliferation in RC-4 B/C cells. Its effect on cell cycle is based partly on downregulation of survivin and partly on survivin-independent inhibition of cyclin A and CDK2. ASA had no effect on pituitary cell apoptosis. Our results suggest that survivin inhibitors and ASA could be potential therapeutic agents for nonfunctioning pituitary adenomas.

## MATERIALS AND METHODS

### Tissue samples

A total of 66 patients diagnosed with pituitary adenoma, comprising 45 nonfunctioning and 21 GH-secreting adenomas, were selected for this study (Table 1). Pituitary adenoma tissues were surgically removed at the National Institute of Clinical Neurosciences, Budapest, Hungary between 2007 and 2017. Histological diagnoses were performed at the 1^st^ Department of Pathology and Experimental Cancer Research, Semmelweis University, Budapest, Hungary according to the WHO classification [32]. The clinical diagnosis of adenomas was based on the hormone levels measured in serum of the patients, and on the histological diagnosis including hormone expressions. Ten normal pituitary samples for mRNA expression analysis were obtained by autopsy within 6 h of death from patients with no evidence of any endocrine disease (University Clinical Centre, Belgrade, Serbia). For immunostaining five normal pituitary samples adjacent to pituitary adenomas were used from formalin-fixed paraffin-embedded (FFPE) tissue blocks.

**Table 1:** Sample characteristics

Experiment	Clinical diagnosis	Sex	Age	Immunhistochemistry for anterior lobe hormones	Tumor size based on preoperative MRI (mm^3^)	Ki 67 proliferation index (%)
IHC	Nonfunctioning	M	38	FSH, LH	*NA*	3.5
IHC	Nonfunctioning	M	51	FSH	*NA*	2.5
IHC	Nonfunctioning	F	83	FSH	*NA*	<2
IHC	Nonfunctioning	M	47	FSH, LH	*NA*	2.5
IHC	Nonfunctioning	F	58	Negative	*NA*	2.5
IHC	Nonfunctioning	M	72	Negative	*NA*	2.5
IHC	Nonfunctioning	M	73	FSH, LH	12635	2.5
IHC	Nonfunctioning	M	44	FSH, LH	4284	3.5
IHC	Nonfunctioning	F	53	FSH	*NA*	5
IHC	Nonfunctioning	F	73	FSH, LH	12558	3.5
IHC	Nonfunctioning	M	44	FSH	4554	2
IHC	Nonfunctioning	M	76	FSH, LH	*NA*	<3
IHC	Nonfunctioning	M	77	LH, TSH, PRL	*NA*	3
IHC	Nonfunctioning	M	62	FSH, LH	*NA*	5
IHC	Nonfunctioning	M	48	FSH, LH	*NA*	<3
IHC	Nonfunctioning	M	52	FSH, LH	*NA*	3
IHC	GH-producing	F	60	GH, PRL	*NA*	<3
IHC	GH-producing	M	22	GH, PRL	11571	3
IHC	GH-producing	M	35	GH, PRL	*NA*	6
IHC	GH-producing	M	49	GH	*NA*	<1
IHC	GH-producing	F	22	GH, PRL	*NA*	4.5
IHC	GH-producing	F	37	GH	*NA*	3
IHC	GH-producing	F	48	GH, PRL	3366	10
IHC	GH-producing	F	43	GH	*NA*	2
IHC	GH-producing	M	35	GH, PRL	*NA*	4
RT-qPCR	Nonfunctioning	F	46	Negative	31350	0
RT-qPCR	Nonfunctioning	F	65	Negative	127.4	2.5
RT-qPCR	Nonfunctioning	F	70	Negative	15000	0
RT-qPCR	Nonfunctioning	F	71	*NA*	12000	0
RT-qPCR	Nonfunctioning	M	55	Negative	4821	0.6
RT-qPCR	Nonfunctioning	M	36	ACTH	1750	0
RT-qPCR	Nonfunctioning	F	29	FSH	6000	0
RT-qPCR	Nonfunctioning	F	55	Negative	11592	2
RT-qPCR	Nonfunctioning	M	58	PRL	5130	0
RT-qPCR	Nonfunctioning	F	75	FSH	8400	0
RT-qPCR	Nonfunctioning	M	34	FSH	26250	0
RT-qPCR	Nonfunctioning	M	69	*NA*	11700	*NA*
RT-qPCR	Nonfunctioning	M	74	Negative	26400	3
RT-qPCR	Nonfunctioning	F	55	Negative	*NA*	2
RT-qPCR	Nonfunctioning	M	73	Negative	*NA*	1.5
RT-qPCR	Nonfunctioning	F	40	Negative	19344	2
RT-qPCR	Nonfunctioning	F	31	Negative	*NA*	4
RT-qPCR	Nonfunctioning	M	80	Negative	*NA*	1
RT-qPCR	Nonfunctioning	F	43	Negative	*NA*	0
RT-qPCR	Nonfunctioning	M	50	Negative	*NA*	2
RT-qPCR	Nonfunctioning	M	61	Negative	12690	3
RT-qPCR	Nonfunctioning	M	53	Negative	*NA*	3
RT-qPCR	Nonfunctioning	F	68	Negative	*NA*	2
RT-qPCR	Nonfunctioning	M	72	Negative	4420	1
RT-qPCR	Nonfunctioning	M	55	FSH, LH, TSH	21952	<2
RT-qPCR	Nonfunctioning	F	48	Negative	*NA*	<2
RT-qPCR	Nonfunctioning	F	60	Negative	*NA*	1
RT-qPCR	Nonfunctioning	F	50	FSH	1200	0
RT-qPCR	Nonfunctioning	M	77	*NA*	7200	*NA*
RT-qPCR	GH-producing	F	59	GH	11616	2
RT-qPCR	GH-producing	F	56	Negative	4800	0
RT-qPCR	GH-producing	M	56	GH	1960	3
RT-qPCR	GH-producing	M	51	GH	*NA*	1.5
RT-qPCR	GH-producing	M	38	GH	*NA*	3
RT-qPCR	GH-producing	F	61	Negative	6624	1
RT-qPCR	GH-producing	F	69	GH, FSH, LH, TSH	*NA*	<1
RT-qPCR	GH-producing	M	37	PRL, GH	2160	*NA*
RT-qPCR	GH-producing	F	52	PRL, GH, FSH, LH, TSH	960	4
RT-qPCR	GH-producing	M	20	PRL, GH	14000	4.5
RT-qPCR	GH-producing	M	55	PRL, GH	90	0
RT-qPCR	GH-produNPcing	F	53	Negative	5355	0

Abbreviations: F: female, FSH: follicle-stimulating hormone, GH: growth hormone, LH: luteinizing hormone, M: male, NA: not available, NFPA: nonfunctioning pituitary adenoma, PRL: prolactin, RT-qPCR: quantitative reverse transcription PCR.

The study was approved by the Scientific and Research Committee of the Medical Research Council of Hungary (0618/15), and the samples were obtained after acquiring written informed consent from all patients.

### Survivin immunohistochemistry on pituitary adenoma

FFPE tissue sections of 16 NFPAs, 9 GH-producing pituitary adenomas, 5 normal adenohypophysis and a lymph node as positive control were selected for immunohistochemical analysis. 4μm-thick sections were immunostained following standard procedure as we previously described [6]. Anti-survivin antibody (71G4B7, #2808, Cell Signaling Technology, ZA, Leiden, The Netherlands) was used at 1:4000 dilution. Goat anti-rabbit immunoglobulin (#P0448, Agilent, Santa Clara, CA, USA, 1:200) was applied as secondary antibody. Slides were developed with DAB (Novocastra Laboratories) and counterstained with haematoxylin. The stained slides were digitally scanned with a high-resolution scanner (Pannoramic Scan, 3DHISTECH Ltd.), and used for virtual microscopic evaluation and quantification with NuclearQuant module of the CaseViewer v2.1 software (3DHISTECH Ltd.). Survivin staining was scored negative or weak, medium or strong positive (see Supplementary Figure 3), and the percentage of nuclei with each staining intensity per case were calculated by the software. Medium and strong staining was used for analysis in comparison of adenoma and normal tissues.

### Cell culture and drug treatment

RC-4 B/C (CRL-1903) and GH3 (CCL-82.1) cells were purchased from American Type Culture Collection (ATCC). Cells were cultured following ATCC recommendations and were used for experiments for not more than 20 passages. Acetylsalicylic acid (aspirin, ASA) was purchased from Sigma Chemical Co. (#A5376, St. Louis, MO, USA), and YM155 was obtained from Selleckchem (#S1130, Munich, Germany). Stock solutions of both compounds were prepared in dimethyl sulfoxide (DMSO). Human Recombinant TRAIL was purchased from Thermo Fisher Scientific (Waltham, MA, USA) and dissolved in nuclease-free water containing 0.1% BSA according to the manufacturer’s instructions. For cell treatment TRAIL was used in 2 ug/ml final concentration.

### Protein extraction and western blotting

Protein was extracted as we previously published [6], and the concentration was determined by BCA assay (Sigma, St. Louis, MO, USA). Total protein was separated by 10-15% SDS polyacrylamide gel electrophoresis, transferred to a PVDF membrane and incubated overnight with primary antibodies (survivin (1:500; #2808), CDK2 (1:1000; #2546), p-CDK2 (1:1000; #2561) from Cell Signaling and Cyclin A2 (1:1000; #MA1-154) from Thermo Fisher Scientific). For loading control membranes were stripped and re-probed using mouse anti-β-actin (1:2000, Cell Signalling Technology, ZA, Leiden, The Netherlands). Anti-mouse and anti-rabbit HRP-conjugated IgGs were used as secondary antibodies (1:2000, #P044701, #P044801 Agilent, Santa Clara, CA, USA). Band intensities were quantified using Image J software (Bethesda, MD, USA).

### Viability and proliferation assay

Cell viability and proliferation was assessed by Alamar Blue assay (#DAL1025, Thermo Fisher Scientific, Waltham, MA, USA). AlamarBlue indicates cell growth by detecting metabolic activity of cells therefore it gives information about the sum of cell growth/cell number and cell viability. Cells were seeded in a 96-well plate 24 hours before treatment. After the indicated treatment 10% Alamar Blue was added to cells and incubated at 37°C for 1h. Fluorescence measurement (ex: 560 nm; em: 590 nm) was performed using a Thermo Scientific Varioskan Flash plate reader (Thermo Fisher Scientific, Waltham, MA, USA). Cell growth was assessed by counting of live and dead cell number in order to verify that the alterations were not due to cell viability changes.

### Cell cycle analysis

After treatment cells were washed with PBS, trypsinized, and pelleted. The supernatant was removed and the cells were fixed overnight in 70% ethanol at -20°C. Before measurement fixed cells were centrifuged for 5 min at 1600 rpm and the pellet was resuspended in extraction buffer (200 mM Na_2_HPO_4_, pH=7.8) supplemented with 10 ug/ml RNase A. After 15 min incubation at room temperature propidium iodide solution was added to the mix (10 ng/ml final concentration) and incubated for another 15 min at room temperature. At least 10000 events were measured from each sample using a FACSCalibur flow cytometer (BD Biosciences, San Jose, CA, USA). The cell cycle distribution of cells and the level of apoptosis were determined using ModFit and Cell Quest Pro software.

### Survivin plasmid and siRNA

To generate survivin-overexpressing cells, they were transfected with pcDNA3.1-Birc5 using X-tremeGENE HP DNA Transfection Reagent (Sigma, St. Louis, MO, USA). The expression level was determined by immunoblot analysis at 48 hours after transfection. Transfection efficiency was 80%, as evaluated by transfection of a GFP-containing plasmid. For survivin silencing, cells were transfected with 2 different Locked Nucleic Acid small interfering RNAs (siRNAs) against survivin (Silencer Select s133761, s133762, Thermo Fisher Scientific, Waltham, MA, USA) or a negative control siRNA using Lipofectamine RNAiMAX (Thermo Fisher Scientific, Waltham, MA, USA). All siRNAs were used in 10 nM final concentration. Gene knockdown was verified by immunoblot analysis.

### Gene expression profiling by TaqMan Low Density Array (TLDA)

Expression changes of apoptosis genes were measured in 29 nonfunctioning and 12 GH-producing pituitary adenomas versus 10 normal pituitary tissues with TaqMan Low Density Array (TLDA) (Thermo Fisher Scientific, Waltham, MA, USA). Tissue samples were stabilized in RNAlater (#AM7020, Thermo Fisher Scientific, Waltham, MA, USA) and stored at -70°C until total RNA was isolated using miRNeasy Mini Kit (#217004, Qiagen, Hilden, Germany). After reverse transcription of 1 ug RNA of each samples with Superscript III First Strand Synthesis Kit (Thermo Fisher Scientific, Waltham, MA, USA), 5 ul product was mixed with 55 ul TaqMan Universal PCR Master Mix (Thermo Fisher Scientific, Waltham, MA, USA) and 50ul nuclease-free water. 100 ul mix was loaded into each channel of the TLDA cards. RT-qPCR reaction was performed using 7900 Fast Real-Time PCR System (Thermo Fisher Scientific, Waltham, MA, USA). (Supplementary Table 1).

### Individual gene expression measurements using RT-qPCR

Total RNA was isolated from cells using RNeasy Mini Kit (#74104, Qiagen, Hilden, Germany). RNA concentration was measured by NanoDrop 1000 Spectrophotometer (Thermo Fisher Scientific, Waltham, MA, USA). RNA samples were reverse transcribed using High-Capacity cDNA Reverse Transcription Kit (Thermo Fisher Scientific, Waltham, MA, USA) according to the manufacturer’s instructions.

Survivin gene expression was measured using predesigned TaqMan gene expression assay (rno-BIRC5: Rn00574012_m1, hsa-BIRC5: Hs04194392_s1, hsa-ACTB: Hs99999903_m1, 18S: Hs99999901_s1) and TaqMan Universal Master Mix (Thermo Fisher Scientific, Waltham, MA, USA).

CCNA2 and CDK2 gene expression were measured using PowerUp SYBR Green Master Mix (#A25742, Thermo Fisher Scientific, Waltham, MA, USA) and custom designed primers (rno-CCNA2 forward: GGATGGTAGTTTTGAATCACCCC, reverse: GGATGGCCCGCATACTGTTA, rno-CDK2 forward: GCTTATCAACGCAGAGGGGT and reverse: GGGTCACCATTTCGGCAAAG, rno-ACTB forward: AGATCAAGATCATTGCTCCTCCT and reverse: ACGCAGCTCAGTAACAGTCC).

All measurements were performed in triplicates on 384-well plates using a Quant Studio 7 Flex Real-Time PCR Systems (Thermo Fisher Scientific, Waltham, MA, USA). The expression level was calculated by the ddCt method.

### Construction of Birc5 expressing plasmid

The sequence of rat *Birc5* gene was confirmed by bidirectional Sanger sequencing. Genomic DNA was isolated from RC-4 B/C rat pituitary adenoma cells using QIAamp DNA Mini Kit (#51304, Qiagen, Hilden, Germany), and the coding sequence was PCR-amplified using the following custom-made primers obtained from Integrated DNA Technologies. Sequences 5’-3’: BIRC5-ex1F: CGGAAGGCGACTTTTTCCAG; BIRC5-ex1R: TGTGTATGAACGCCGAGGTG; BIRC5-ex2F: CTCTCGGCCCGGAAAGATT; BIRC5-ex2R: TCCAGTTCTTCCCAAAAGACTCC; BIRC5-ex3F: AAAGACAGCCGTGGAGATGG; BIRC5-ex3R: TCCCTGAGACAGATCCCCAG; BIRC5-ex4F: CTCCCTTGGTAGGCGAGC; BIRC5-ex4R: CGGTCTCCTGTAAGACACCAA.

Rat cDNA library was prepared from the mRNA of RC-4 B/C rat pituitary adenoma cells using High-Capacity cDNA Reverse Transcription Kit (#4368813, Thermo Fisher Scientific, Waltham, MA, USA), and served as a template for insert amplification (Sequences 5’-3’: forward primer: CGAAGCTTCACCATGGGTGCTCCGGCGCTGC and reverse primer: CGTCTAGAGTCAGCGTAAGGCAGCC AGCTG) using High Fidelity DNA Polymerase (#M0530, New England Biolabs, Ipswich, MA, USA). The insert was subcloned into a pcDNA3.1 vector (Thermo Fisher Scientific, Waltham, MA, USA) using XbaI and HindIII-HF restriction enzymes (#R0145 and #R3104, New England Biolabs, Ipswich, MA, USA). Ligation was performed using T4 DNA ligase (#M0202, New England Biolabs, Ipswich, MA, USA) at 16°C overnight. After heat shock transformation of chemical competent *Escherichia coli* DH5α, bacteria were plated on ampicillin-containing LB agar and grown at 37°C overnight. Next day colonies were checked and two of the positive clones were separately grown in liquid LB at 37°C overnight. For isolation of recombinant DNA PureLink HiPure Plasmid Midiprep Kit (#K2100-03, Thermo Fisher Scientific, Waltham, MA, USA) was used. Before transfection, the sequence of the construct was confirmed with bidirectional Sanger sequencing using the following primers (5’-3’) located on the vector: forward primer: AGAACCCACTGCTTACTGGC, reverse primer: GGCAAACAACAGATGGCTGG.

### Caspase-3 apoptosis assay

Caspase-3 activity of cells was determined using a Caspase-3 Colorimetric Protease Assay Kit (#KHZ0021, Thermo Fisher Scientific, Waltham, MA, USA) according to the manufacturer’s instructions. Briefly, after 72 hours of treatment, cells were re-suspended in chilled cell lysis buffer and incubated on ice for 10 min. After 1 min centrifugation at 10000 x g, protein concentrations of the supernatants (cytosol extract) were measured using BCA Protein Assay Kit (#B6916, Sigma, St. Louis, MO, USA). Equal amounts of proteins were then incubated at 37°C with Reaction Buffer containing 10 mM DTT and 200 uM DEVD-pNA substrate for 2 hours. The optical density was measured at 405 nm with a Thermo Scientific Varioskan Flash plate reader (Thermo Fisher Scientific, Waltham, MA, USA).

### Trypan blue exclusion assay

Cells were seeded in a 6-well plate 24 hours before treatment. After the indicated treatment cells were stained with Trypan blue (#15250, Thermo Fisher Scientific, Waltham, MA, USA), which can be taken up only by viable cells. Viable and dead cells were counted using a Burker’s chamber.

### DNA fragmentation assay

DNA degradation is a key indicating factor of the presence of either apoptosis or necrosis. For detection of cell death by DNA fragmentation assay, cells were seeded in a 6-well plate 24 hours before treatment. After treatment DNA was isolated from cells using QIAamp DNA Mini Kit (#51304, Qiagen, Hilden, Germany) and analyzed on 1% agarose gel containing ethidium-bromide.

### Xenograft experiment

Inbred SCID mice were used for generating GH3 and RC-4 B/C xenograft models. Experiments were carried out in accordance with the animal protection laws of the Ethic Committee of Semmelweis University. 6-8-week-old male mice were subcutaneously injected with GH3 and RC-4 B/C cells mixed with Matrigel Matrix (#354262, Corning) in one flank. Tumor size was measured once a week using calipers, and tumor volume was calculated as width^2^ × length × 0.5. As RC-4 B/C xenograft has been never generated before, we performed xenograft injection with gradually increasing cell number (5x10^6^, 10x10^6^ and 20x10^6^/injection). We used two mice in every experiment and repeated it three times. As a positive control we also injected additional mice with GH3 cells (10x10^6^) as positive control.

### Statistical analysis

Statistical analysis was performed using GraphPad Prism 6 software (GraphPad Software Inc., La Jolla California USA). For examining the differences in cell viability, proliferation, cell-cycle distribution, mRNA and protein expression one-way ANOVA was used followed by Tukey’s multiple comparisons test. Investigating correlation between survivin expression and Ki67 index Spearman correlation was used. For comparison of staining scores between adenoma and normal tissues Welch's unequal variances t-test was used. Fisher’s exact test was applied to compare the prevalence of moderate and strong positivity between adenoma and normal samples. P-values < 0.05 were considered significant.

## SUPPLEMENTARY MATERIALS FIGURES AND TABLE



## Abbreviations

AKT2: AKT Serine/Threonine Kinase 2
ASA: Acetylsalicylic acid
AURKB: Aurora Kinase B
BAG1: BCL2 Associated Athanogene 1
BAX: BCL2 Associated X, Apoptosis Regulator
BCL-2: BCL2 Apoptosis Regulator
BIRC5: Baculoviral IAP Repeat Containing 5, survivin
CCNA1, A2, B1, B2: Cyclin A1, A2, B1, B2
CDC25A: Cell Division Cycle 25A
CDK2: Cyclin Dependent Kinase 2
CDKN1A: Cyclin Dependent Kinase Inhibitor 1A
CDKN1B: Cyclin Dependent Kinase Inhibitor 1B
CDKN2A: Cyclin Dependent Kinase Inhibitor 2A
DAPK: Death Associated Protein Kinase 1
E2F1: E2F Transcription Factor 1
GADD45G: Growth Arrest and DNA Damage Inducible Gamma
GAL3: Galectin 3
GH: Growth hormone
HMGA1/2: High Mobility Group AT-Hook 1/2
IAP: Inhibitor of Apoptosis Protein
INCENP: Inner Centromere Protein
MEG3: Maternally Expressed Gene 3
NFPA: Nonfunctioning pituitary adenoma
NSAID: Nonsteroidal anti-inflammatory drug
PLAGL1: Pleiomorphic Adenoma Gene-Like 1
POUF1: POU Class 1 Homeobox 1
PTTG: Pituitary Tumor Transforming Gene
SCID: Severe combined immunodeficient
SP1: Sp1 Transcription Factor
TRAIL: TNF-related apoptosis-inducing ligand
WEE1: WEE1 G2 Checkpoint Kinase
