# Supplementary material for: Survivin as a potential therapeutic target of acetylsalicylic acid in pituitary adenomas
Source: Oncotarget. 2018 Jun 26;9(49):29180–92. doi: 10.18632/oncotarget.25650 (PMC6044388; doi:10.18632/oncotarget.25650)
Supplement: Supplementary file 1 [file oncotarget-09-29180-s001.pdf]

Survivin as a potential therapeutic target of acetylsalicylic acid in pituitary adenomas

SUPPLEMENTARY MATERIALS

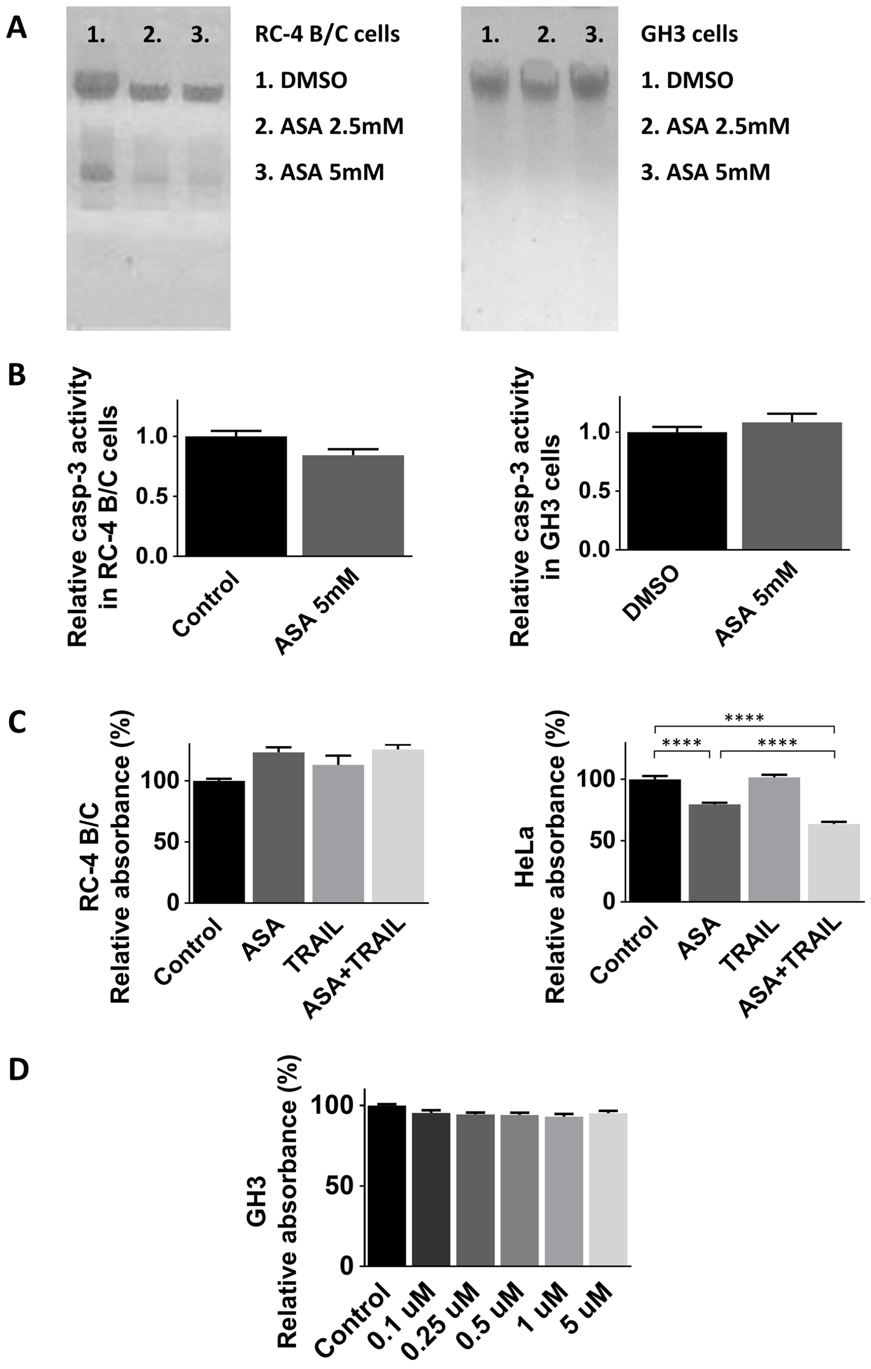

Supplementary Figure 1: ASA does not induce apoptosis in pituitary adenoma cells. **(A)** DNA degradation assay shows no apoptosis induction in RC-4 B/C and GH3 cells. **(B)** ASA did not increase Caspase-3 activity in pituitary adenoma cells. **(C)** ASA did not induce TRAIL-mediated apoptosis in pituitary adenoma cells compared to a positive control (HeLa cells) ****: $p<0.0001$. **(D)** YM155 did not change the proliferation of GH3 cells.

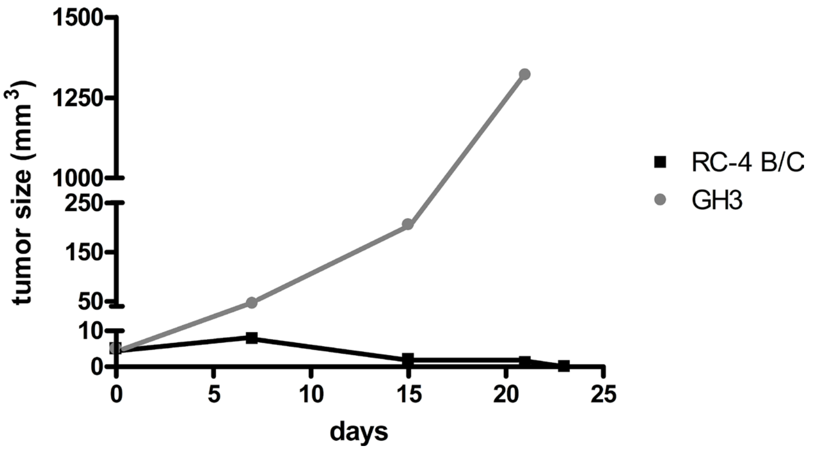

Supplementary Figure 2: Growth curves of RC-4 B/C and GH3 xenografts in SCID mice. GH3 cells formed palpable tumor after the first two weeks and started to grow rapidly. In contrast RC-4 B/C cells were not able to adhere and form xenograft tumor, and after two weeks they were progressively absorbed.

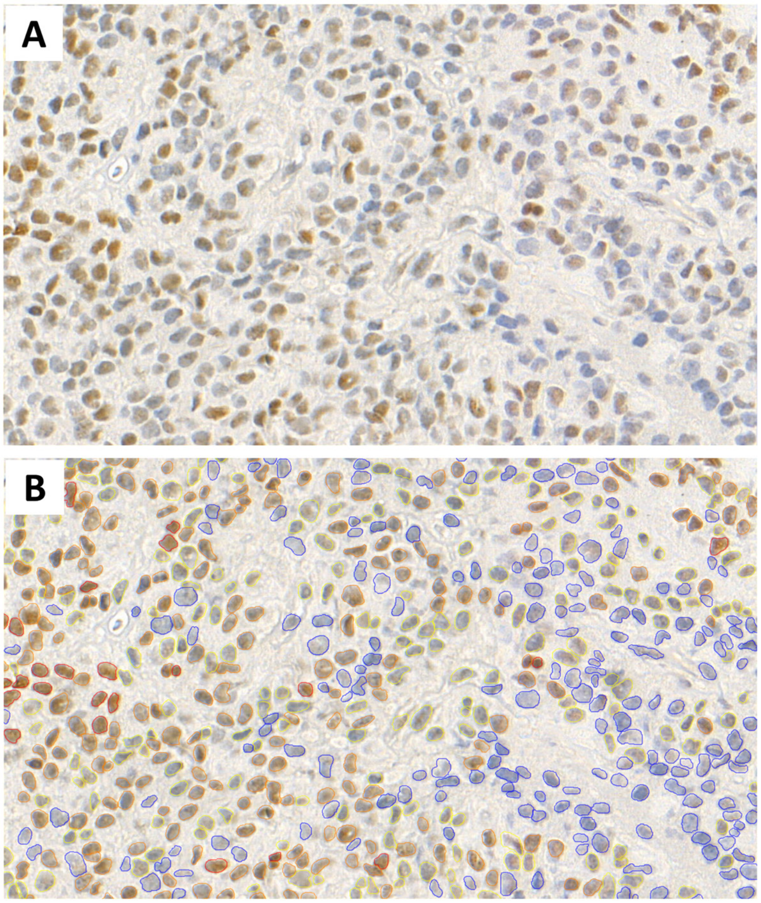

Supplementary Figure 3: Representative images used for scoring survivin immunostaining with the NuclearQuant module of the CaseViewer v2.1 software (3DHISTECH Ltd.). **(A)** Representative area of a pituitary adenoma with negative cells, as well as with weak, medium and strong nuclear survivin positivity. **(B)** The same area as shown in A, with blue, yellow, orange and red circles around the nuclei as highlighted by the software, representing negativity, and weak, medium and strong positivity, respectively (400x magnification).

Supplementary Table 1: Assays used for gene expression profiling of pituitary adenomas by TaqMan Low Density Array (TLDA)

Gene Name	Gene Symbol	Assay ID
Tumor protein p53	TP53	Hs01034249_m1
Bcl-2-associated death promoter	BAD	Hs00188930_m1
Bcl-2 homologous antagonist/killer	BAK1	Hs00832876_g1
Bcl-2-associated X protein	BAX	Hs00180269_m1
BCL-2 Associated Athanogene 1	BAG1	Hs00185390_m1
Apoptosis Regulator Bcl-2	BCL2	Hs00608023_m1
Bcl-2-Like Protein 1	BCL2L1	Hs00236329_m1
BCL2-Like 11 (Apoptosis Facilitator)	BCL2L11	Hs00708019_s1
BCL2 Binding Component 3	BBC3	Hs00248075_m1
BH3 Interacting Domain Death Agonist	BID	Hs00609632_m1
Tumor Necrosis Factor (Ligand) Superfamily, Member 10; TNF-Related Apoptosis Inducing Ligand TRAIL	TNFSF10	Hs00921974_m1
X-Linked Inhibitor Of Apoptosis	XIAP	Hs00745222_s1
Apoptotic Peptidase Activating Factor 1	APAF1	Hs00559441_m1
Cytochrome C, Somatic	CYCS	Hs01588974_g1
Phosphatase And Tensin Homolog	PTEN	Hs02621230_s1
Nuclear Factor Kappa B Subunit 2	NFKB2	Hs00174517_m1
JunD Proto-Oncogene, AP-1 Transcription Factor Subunit	JUND	Hs02330233_u1
